# Midterm clinical and radiological outcomes of arthrogryposis-associated clubfoot treated with the Ponseti method: a retrospective observational study and comprehensive literature review

**DOI:** 10.1186/s13018-024-05101-3

**Published:** 2024-09-28

**Authors:** Nina Berger, Hans Forkl, Bernhard Heimkes, Vincent Frimberger, Ferdinand Wagner, Frank Hildebrand, Heide Delbrück

**Affiliations:** 1 Department of Neuromuscular and Paediatric Orthopaedics, Klinikum Dritter Orden München – Nymphenburg, Menzinger Strasse 44, 80638 München, Germany; 2 Paediatric Orthopaedic Department, Kind im Zentrum Chiemgau, Bernauer Straße 18, 83229 Aschau i, Chiemgau, Germany; 3 Department of Orthopaedics and Traumatology, Klinikum Passau, Innstraße 76, 94032 Passau, Germany; 4 Department of Orthopaedics, Trauma and Reconstructive Surgery, University Hospital RWTH Aachen, Pauwelsstrasse 30, 52074 Aachen, Germany

**Keywords:** Arthrogryposis, Clubfoot, Ponseti

## Abstract

**Background:**

The treatment results of the Ponseti method for arthrogrypotic clubfoot have been described in only a few case series. Further evaluations are necessary.

**Methods:**

Children from two German paediatric orthopaedic hospitals with arthrogryposis-associated clubfoot treated with the Ponseti method between 2004 and 2011 and who were at least five years of age at their last follow-up were retrospectively evaluated. The endpoints were the clinical foot position, necessary surgeries during the follow-up period and radiological constellations. A comprehensive literature review was conducted after a systematic literature search.

**Results:**

Seventeen patients (47% with amyoplasia [AP] and 53% with distal arthrogryposis [DA]) met the inclusion criteria. Thirty-one feet were evaluated. The period between the treatment start and the last follow-up examination covered 8.9 ± 2.5 years. After the last cast removal within the initial Ponseti cast series, 74% of the clinical results were good to excellent. However, the clinical outcomes in the patients with AP were significantly worse. Overall, in 23 feet (74%), at least one major surgery at the age of 2.9 ± 2.2 years was necessary during the clinical course. Major surgeries were much more frequent on the feet of the patients with AP than with DA. Lateral X-rays showed normal age-appropriate radiological angles in 4% of the feet, hindfoot equinus in 19%, under-corrected hindfoot in 44%, under-corrected clubfoot in 26% and rocker bottom deformity in 7%. The radiological residual deformities in AP were much more severe than in DA (*p* = 0.042). Most of the studies reviewed (11 case series, 144 patients) reported high initial clinical correction rates, followed by high recurrence rates and the need for further surgeries.

**Conclusion:**

About a quarter of the arthrogrypotic patients benefited from the Ponseti therapy without further major surgery. However, the clinically observed high initial correction rate after Ponseti therapy of arthrogrypotic clubfoot was not accompanied by a correction of the bony foot position in the X-rays. The feet of the patients with DA had better outcomes than those of the patients with AP. Therefore, in outcome studies, a clear distinction between patients with AP and those with DA is necessary.

**Supplementary Information:**

The online version contains supplementary material available at 10.1186/s13018-024-05101-3.

## Introduction

Arthrogryposis multiplex congenita (AMC) refers to a group of congenital conditions characterised by joint contractures in two or more body areas [1]. It is considered a rare disease that occurs in 1 out of 3,337 (Helsinki, Finland) to 1 out of 12,037 (Western Australia) live births [2]. Multiple congenital contractures (i.e., arthrogryposes) are due to amyoplasia (AP), distal arthrogryposis (DA) or syndromal diseases [3].

In AP, all four limbs are usually affected. It is assumed to occur sporadically and to be uninherited. AP patients are severely affected and physically limited, and require support in their daily activities. Typical clinical pictures of these patients show atrophied muscles of extremities with internally rotated shoulders, extended elbows, flexed and ulnary deviated wrists, stiff fingers, dislocated hips, extended knees and an equinovarus position of the feet. DA has a genetic origin, and distal parts of the limbs are primarily affected, but the clinical presentation varies [3, 4]. In 2009, Bamshad et al. described ten different types of distal arthrogryposis [3]; and in 2024, Illés et al. described twelve [5].

Arthrogryposis is often accompanied by a clubfoot. Sonographic findings in antenatally detected cases found a clubfoot in 83% of cases [6].

A largely physiological foot position or at least feet that are suitable for orthotic treatment are of utmost importance for learning to walk and, thus, for the development of the child. The aim of clubfoot treatment in AMC is a plantigrade foot. The treatment of arthrogrypotic clubfoot has always been considered difficult, as the feet are particularly stiff and often shorter than normal [7]. To correct this condition, extensive soft tissue and bone surgery was performed until the Ponseti method [8] became widespread [9].

In 2008, the first results of the treatment using the Ponseti method for arthrogrypotic clubfoot were published. The method was considered a good option for the initial treatment [10–12].

The aim of the present study was to evaluate the treatment results of arthrogrypotic feet with the Ponseti method, depending on the presence of AP or DA. Furthermore, the frequency of necessary minor and major surgeries during the clinical course and radiological correction results were investigated. These results should be placed in the context of a comprehensive review of recent literature regarding Ponseti-treated arthrogrypotic clubfeet, which considers all case series published to date.

## Methods

The present study was performed according to the Strengthening the Reporting of Observational Studies in Epidemiology (STROBE) guidelines [13]. It was approved by the Ethics Committee of Bayrische Landesärztekammer in Munich, Germany (approval no. 24069).

### Included patients

This study was a retrospective study in two German paediatric orthopaedic centres (Klinikum Dritter Orden Munich and Orthopaedische Kinderklinik Aschau im Chiemgau). Children with arthrogryposis-associated clubfoot (both AP and types 1, 3 and 4 DA) treated with the Ponseti method between 2004 and 2011 were included (Supplementary Table 1). Information on the DA subtype was obtained retrospectively from medical records. The subtype classification was based on the state of knowledge at the time of treatment by clinical examination [3, 14]. A further inclusion criterion was a minimum age of five years at the last follow-up (FU). To find these patients in the aforementioned centres, the medical records of all of their patients with the main diagnosis of clubfoot were evaluated.

### Casting

In all of the children, long leg casts were applied according to a modification of the Ponseti method for complex clubfoot and changed once a week, followed by percutaneous Achilles tenotomy [15]. The initial Pirani score was evaluated before the first cast [16]. After the initial cast series, for the whole day and night, the patients were provided with a standard Denis Browne foot abduction brace [17]. When compliance problems with bracing occurred, the therapy was switched to a circular lower leg orthosis that could be extended to a knee–ankle–foot orthosis design [18]. After the fourth month of life, these orthoses were continued at night. In contrast to the Ponseti method for congenital clubfoot, the children received lower leg orthoses continuously during the day, even after their fourth month of life.

### Endpoints

#### Clinical outcome: foot position and redressability

After the last cast removal within the first Ponseti cast series, the initial correction of the feet was clinically evaluated regarding their forefoot and hindfoot positioning and dorsiflexion ability. The clinical outcomes were classified as follows:

- Excellent clinical outcome: manual over-correction of hindfoot and forefoot was possible with positive dorsiflexion ability;
- Good clinical outcome: manual correction to a neutral position was possible with neutral to positive dorsiflexion;
- Fair clinical outcome: one component of malalignment was not correctable manually, but the foot was, at least, plantigrade; and.
- Poor clinical outcome: more than one component of malalignment was not manually redressable, and the foot could not be placed in the plantigrade position.

#### Necessary surgeries during the follow-up (FU) period

The surgeries were classified into minor and major operations:

- Minor surgeries: Achilles tendon reinterventions, unicompartmental joint procedures (i.e., dorsal arthrolysis), single tendon transfers and extraarticular one-dimensional osteotomies (i.e., calcaneus osteotomies) and.
- Major surgeries: soft tissue multicompartmental joint procedures and corrective and fusion multidimensional bone procedures.

#### Radiologic evaluation

For the radiologic evaluation of the correction results after Ponseti brace therapy, especially, the incidence of rocker bottom foot (RBF) [19], the calcaneus pitch (i.e., calcaneal base angle), the lateral talocalcaneal (latTaCa) angle and the lateral talus-first metatarsal (latTaMT1) angle were measured on the first lateral radiograph taken after the completion of the initial Ponseti cast series under load. The X-ray images were requested by the physician during a regular FU if, in the physician’s opinion, the clinical findings required a radiological examination and the child’s parents agreed to it. These images were used and evaluated as parts of the retrospective evaluation of the patients. An Xray was requested with a board under the sole of the foot and the ankle in a mostly neutral position. The sole of the foot had to be in complete contact with the board, and plantar pressure had to be applied. To evaluate the actual neutral position in the ankle joint, the ventrally opened tibia shaft–sole angle (TSSA) was measured. In this situation, the sole corresponded to the board.

The measured latTaMT1 and latTaCa angles were assessed to age-dependent normal ranges according to Vanderwilde et al. [20], and the calcaneal pitch, according to Davids et al., with a norm of 17 ± 6° [21]. According to Vanderwilde et al., in a foot with a cavus deformity, the latTaMT1 angle will have an increasingly negative value as the severity of the deformity increases, whereas in a flat foot, this angle will become increasingly positive.

The following constellations were defined:

- Normal: normal values of the latTaMT1 angle, latTaCa angle and calcaneal pitch;
- Hindfoot equinus (HE): pathological to small calcaneal pitch;
- Under–corrected hindfoot (UHF): normal latTaMT1 angle, pathological calcaneal pitch and pathological to small latTaCa angle;
- Under–corrected clubfoot (UCF): pathological to small calcaneal pitch, pathological negative latTaMT1 angle and pathological to small latTaCa angle; and.
- Rocker bottom foot (RBF): pathological to small calcaneal pitch, pathological positive latTaMT1 angle and pathological to small latTaCa angle.

### Statistics

The statistical calculations were carried out using IBM SPSS Statistics 29.0.0.0 (IBM, Armonk, NY, US). Fisher’s exact test, the Mann–Whitney U test, linear–by–linear association and Spearman’s correlation were used to evaluate the data. For each measurement type, the significance level was set to 0.05.

### Methods of literature search

On 21 May 2024, a literature search was carried out in PubMed using the search terms ‘clubfoot’, ‘arthrogrypo*’ and ‘Ponseti’. The search words were combined using the Boolean operator ‘AND’. The criteria for a study’s inclusion in the literature review were its reporting of the outcomes of arthrogryposis-associated clubfoot treated with the Ponseti method.

## Results

### Patients

**Fig. 1:**
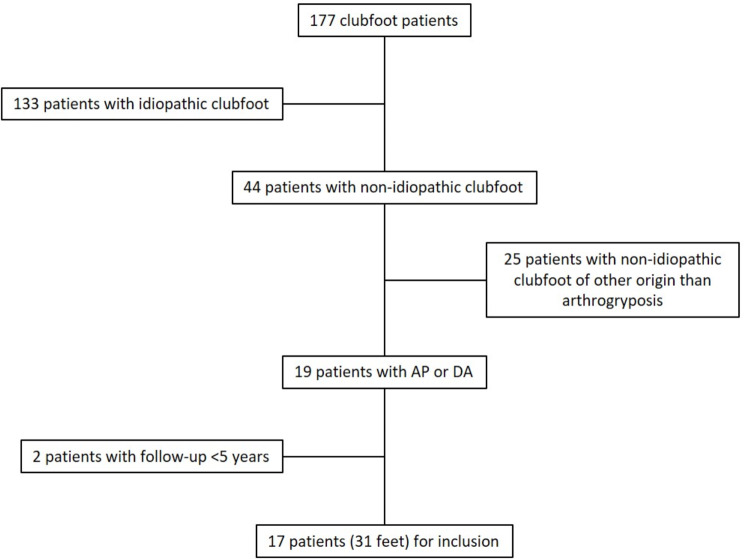
Patient selection

During the study period, 177 patients with clubfoot were treated in both centres. Seventeen of them (31 feet) met the inclusion criteria (Fig. 1). Six of them were female (35%), and 11 were male (65%). Eight had AP (47%), and nine (53%), DA. In seven cases (41%), DA1 was the most frequent DA type. In 14 patients (82%), both feet were affected. The age at the start of the treatment with a cast was 9.0 ± 8.6 (range: 1.3–26.3) weeks, and at the last FU, 9.1 ± 2.4 (range: 5.0–13.1) years. The period between the treatment start and the last FU examination was 8.9 ± 2.5 (range: 4.5–13.0) years.

### Initial cast series

For all 31 feet, the average number of casts was 11.0 ± 3.8 (range: 6–21). Achilles tenotomy was performed during the initial cast series in all of the feet. The last cast was removed at the age of 21.8 ± 8.0 (range: 11.0–41.4) weeks. The Pirani score was 5.5 ± 0.7 (range: 4.0–6.0) points before the first treatment. According to the parents, all of the children, except one, complied with the prescribed orthoses.

### Clinical outcomes

**Fig. 2:**
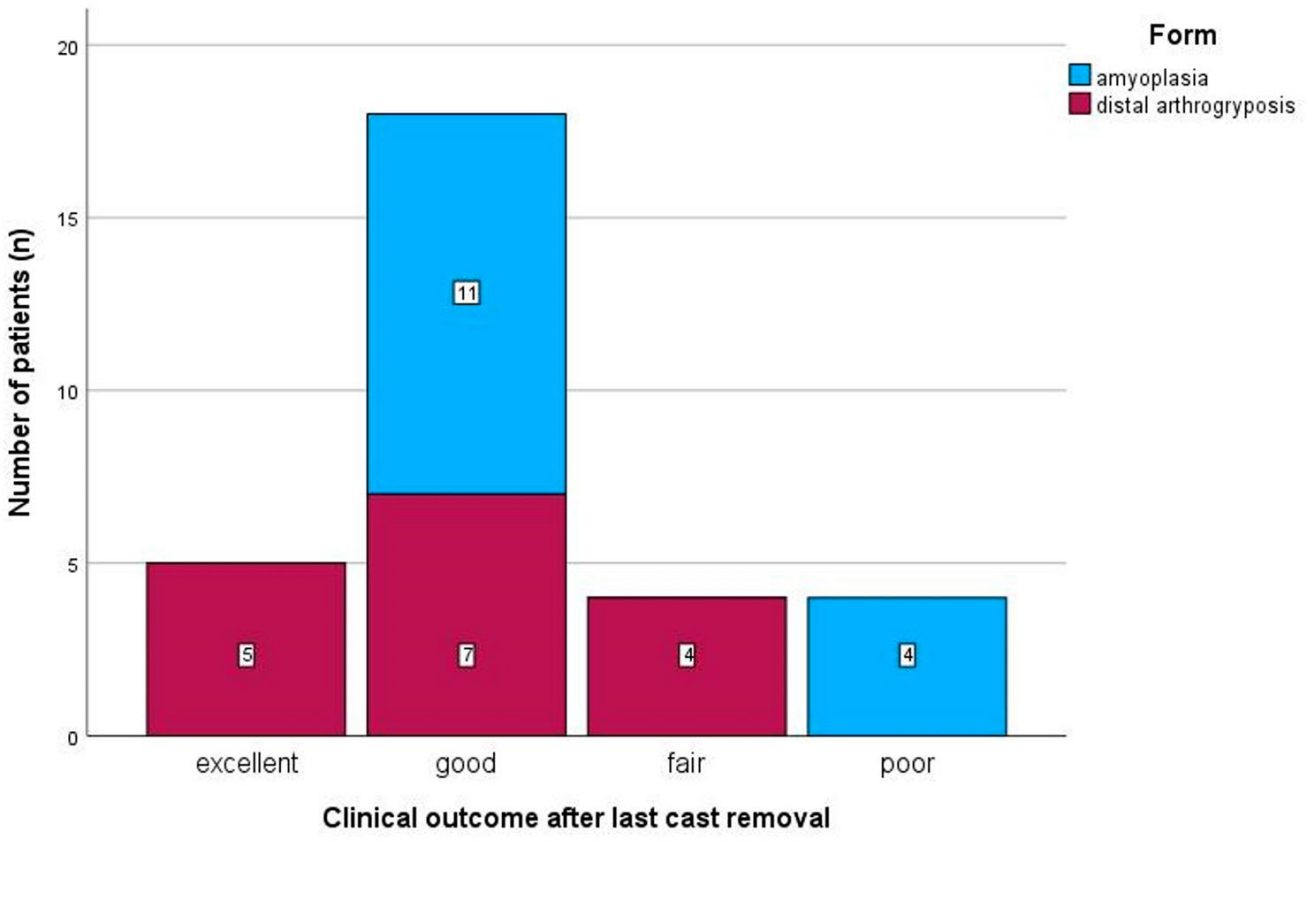
Clinical outcomes at the end of the initial completed Ponseti cast series

After the last cast removal in the initial Ponseti cast series, the observed clinical results were excellent in five feet (16%), good in 18 feet (58%), fair in four feet (13%) and poor in four feet (13%). The clinical outcomes in the patients with AP were significantly worse (*p* < 0.001; Fig. 2).

In all of the feet, the dorsiflexion after the last cast removal was 5.8 ± 7.4° (range: -5–20°). The range of motion (ROM) data were available in the medical records for 26 feet. The ROM at the final FU averaged over the available data was 3.1 ± 3.8° (range: 0–10°) dorsiflexion, 4.4 ± 8.8° (range: 0–30°) neutral position and 10.1 ± 12.8° (range: 0–50°) plantarflexion. The dorsiflexion at the end of the initial cast treatment and the ROM at the last FU did not differ significantly between the patients with AP and those with DA. No patient complained of pain at the last FU.

### Necessary surgeries during the FU period

Up to the last FU, in eight feet (26%), minor surgery (Repeat Achilles tenotomy) after a new cast series was indicated. In one case, dorsal ankle arthrotomy with tibialis anterior tendon transfer was added, and instead of tenotomy, lengthening of the Achilles tendon was carried out. The age at these minor surgeries was 1.2 ± 0.4 (range: 1.0–2.0) years.

In 23 feet (74%), at least one major surgery was necessary during the clinical course—peritalar release in 17 feet (55%) and talectomy and corrective triple arthrodesis (TA) in three feet (10%) each. The age at these first major surgeries was 2.9 ± 2.2 (range: 0.7–8.7) years.

A second major operation had to be performed on six feet (20%): talectomy in three patients (10%), and TA, complex foot reconstruction with supramalleolar derotational osteotomy and TA with supramalleolar derotational osteotomy in one patient (3%) each. The age at the second major surgery was 7.4 ± 2.3 (range: 5.6–11.3) years.

**Fig. 3:**
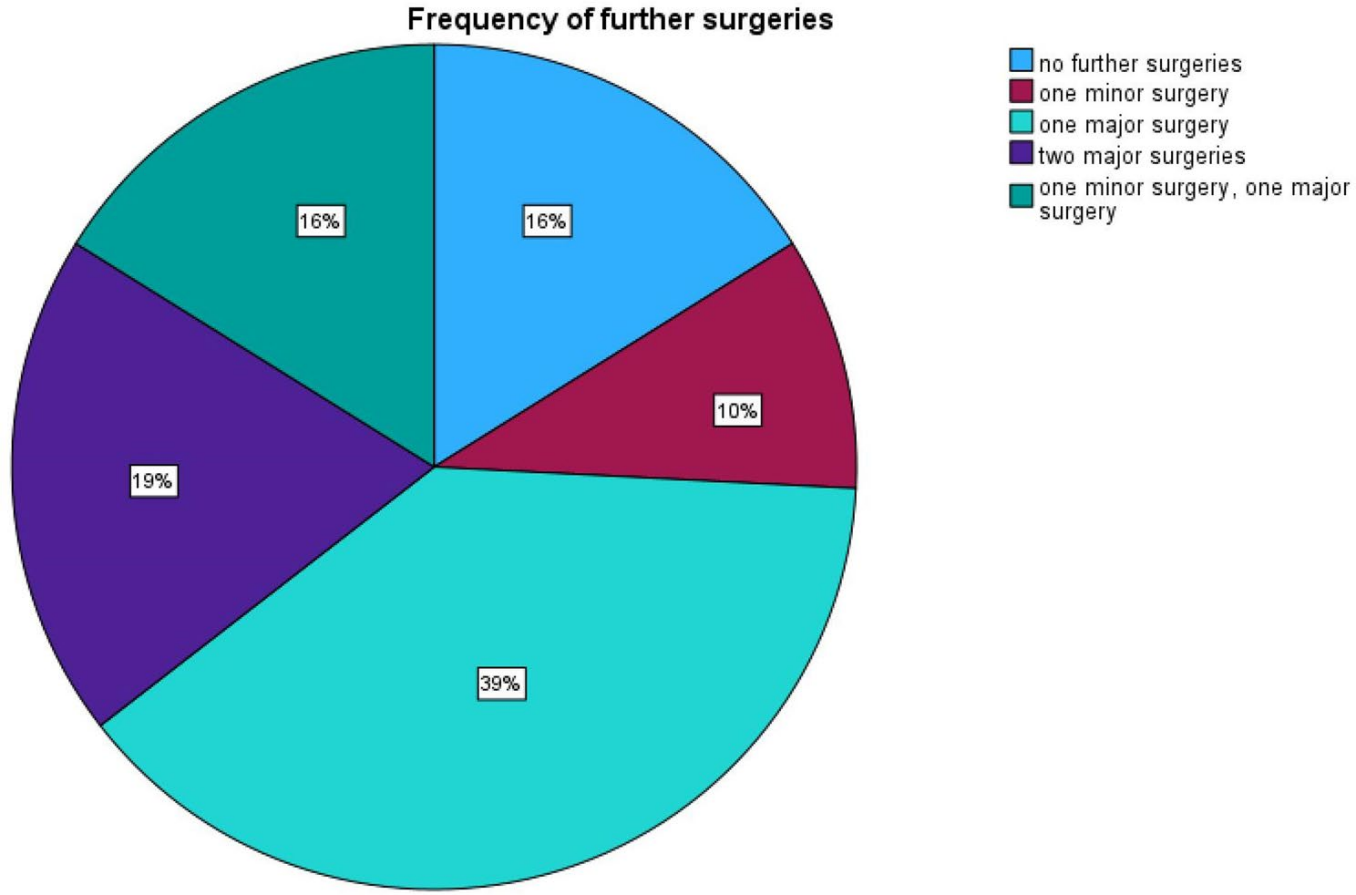
Frequencies of surgeries in the follow-up period

The frequencies of the surgeries on the feet in the FU period are shown in Fig. 3: 16% of the feet needed no further surgery; 10%, one minor surgery; 39%, one major surgery; 19%, two major surgeries; and 16%, one major surgery after a former minor surgery.

**Fig. 4:**
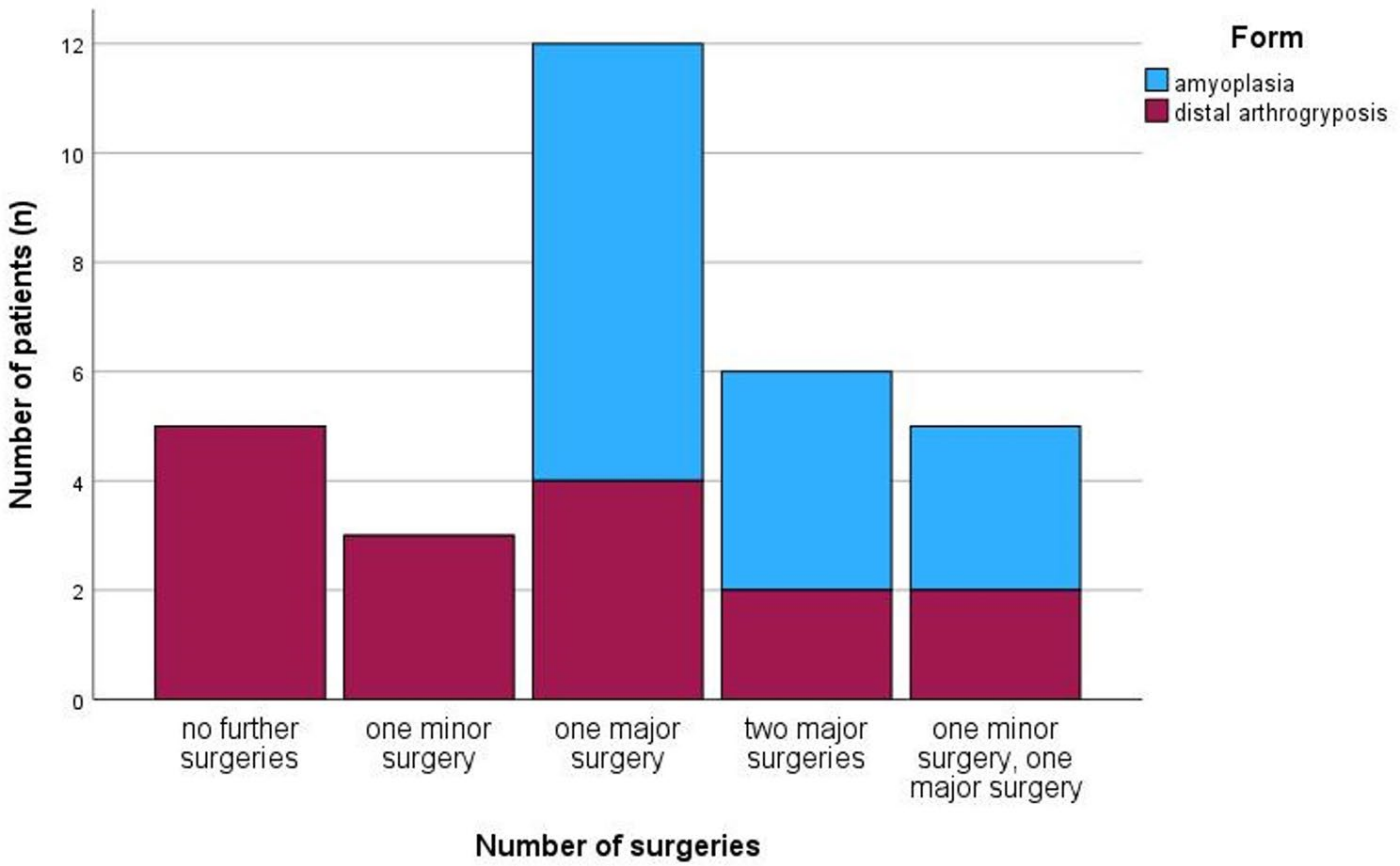
Numbers of surgeries of the patients with amyoplasia and distal arthrogryposis

The frequency of major surgeries was significantly higher on the feet of the patients with AP than with DA (*p* < 0.001). Extended surgeries were needed on all of the feet of the patients with AP but only on eight feet (50%) of the patients with DA (Fig. 4).

The individual patient characteristics, clinical results and further surgeries are shown in Supplementary Table 1.

### Radiological outcomes

In 27 of the 31 feet, X-rays were available after Ponseti casting (Supplementary Table 2). The age at the time of these X-rays was 1.7 ± 1.4 (range: 0.6–6.8) years. The duration of the period between the cast removal and the X-ray was 1.3 ± 1.4 (range: 0–6.5) years. The TSSA was 98 ± 14° (range: 63–132°), indicating that the X-rays with imitation of full weight bearing and 90° TSSA were not always reproducible. The latTaMT1 angle was 7 ± 17° (range: 32–39°); the latTaCa angle, 19 ± 9° (range: 0–38°); and the calcaneal pitch, -11 ± 11° (range: 37–9°).

Normal radiological angles were evaluated in one foot (4%); hindfoot equinus, in five feet (19%); under–corrected hindfoot, in 12 feet (44%); under–corrected clubfoot, in seven feet (26%); and rocker bottom deformity, in two feet (7%).

**Fig. 5:**
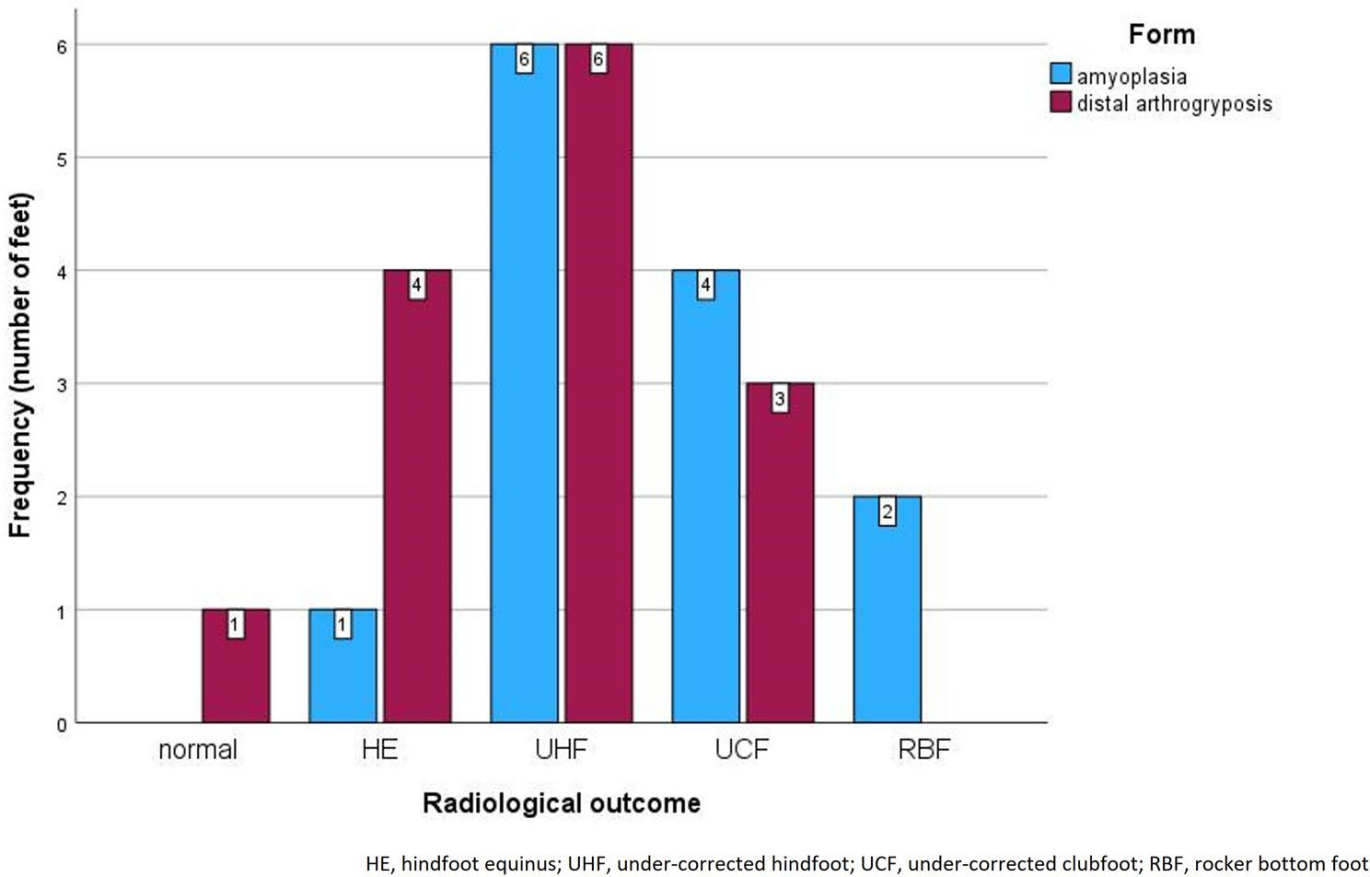
Residual radiological deformity after the first Ponseti cast series

In AP, the residual deformity was significantly more severe (*p* = 0.042; Fig. 5). There was no significant association between the clinical and radiological outcomes (*p* = 0.874). Furthermore, there was no significant Spearman’s correlation between the radiological outcomes and the period from the cast removal to the X-ray (*p* = 0.274).

### Results of the literature search

Table 1 shows the search strategy with the corresponding hits. After abstract and full-text screening of the 27 identified records, 11 case series with reported outcomes after Ponseti treatment of clubfoot in arthrogryposis were selected [10–12, 22–29]. A comprehensive overview of those case series and their outcomes is presented in Supplementary Table 3.

**Table 1:** Search strategy for the comprehensive literature review

	‘clubfoot’	‘arthrogrypo*’	‘Ponseti’
Hits	5170	3232	1099
Hits combined with ‘AND’	27		
Hits with reported outcomes	11		

Overall, the treatment results of the Ponseti method on arthrogrypotic clubfeet of 144 patients were reported. Most of the studies concluded that there were high initial clinical correction rates, followed by high recurrence rates and the need for further surgeries. Most of the authors described the method as suitable for the initial treatment of very young children to delay more extensive surgery. This was also the conclusion of the systematic review and meta-analysis of Bravin et al. [30], which included case series from 2008 to 2016 [10–12, 27, 29].

However, only a few studies explicitly distinguished between children with AP and DA [10, 11, 25, 26, 28, 29]. This aspect was also not considered in the systematic review of Bravin et al. [30]. Radiological results were mentioned for only five patients [11]. The longest FU period was 7.25 (range: 5–10) years [28].

## Discussion

The present study contributes clinical and radiological results of Ponseti therapy in patients with arthrogryposis after the completion of initial cast series and at a mean FU of nearly nine years. Specifically, this study differs from the previous literature because it differentiates the outcomes of patients with AP from those with DA and considers radiological outcomes.

After Ponseti therapy in idiopathic clubfoot, initial correction rates of 90–100% were reported [31]. The systematic analysis of Agarwal et al. of 24 studies and an average FU of six years computed a mean relapse rate of 30% in idiopathic clubfoot [32]. Accordingly, the systematic review and meta-analysis of Bravin et al. of five case series with 102 arthrogrypotic clubfeet in 53 patients found an initial success rate of 91% with the Ponseti method, which fell to 68% after 5.8 years of FU [30].

Compared with these pooled data for idiopathic and arthrogrypotic clubfeet, the results of the present study show higher recurrence and subsequent surgery rates. Excellent and good clinical results were found in 74% of the cases immediately after completion of Ponseti therapy, and 74% of the feet required at least one major surgery during the study period. Only 16% of all of the feet needed no further surgery. A possible important reason for this discrepancy is that in previous studies, AP and DA were not differentiated when the data were pooled; but in the present study, we were able to demonstrate that the initial clinical outcomes were significantly worse—and thus, the need for subsequent operations was greater—in the patients with AP than in those with DA. According to our literature research, we are the first to demonstrate this association. Furthermore, the results of this study emphasise the necessity in the future of detailed consideration of the forms and aetiology of arthrogryposis—made possible by increasing genetic knowledge and modern technologies [33, 34].

Moreover, the FU length seems to correlate positively with the recurrence rate after Ponseti therapy [32]. Based on our comprehensive literature review, so far, the present study reports the longest FU period—8.9 ± 2.5 years—in arthrogrypotic feet after initial Ponseti therapy. This could be a further reason for our higher need for surgeries during the clinical course.

Except for five patients in the study of Kowalczyk et al. [11], there are currently no published radiological FU examinations of patients with Ponseti-treated arthrogrypotic clubfoot. The present study evaluated radiological data from 27 arthrogrypotic feet. Normal radiological angles related to the same age group were found in only one foot (4%). In the systematic literature review of Rastogi et al. of 14 studies that examined 1,122 idiopathic clubfeet, good clinical outcome scores after treatment with the Ponseti method contrasted with the radiological angles, which were mostly outside the normal range [35]. In this context, the age dependence of radiological data on children’s feet should be evaluated, and this was done in the present study, based on the reference values of Vanderwilde et al. [20]. However, it can be assumed that the high recurrence rates in this study can be attributed—at least partially—to the incomplete correction of bone deformities, especially since the bone deformities in the feet of the patients affected by AP were more severe than in the feet of those with DA.

The results of the present study support those of the studies included in the comprehensive literature review herein—that the Ponseti method is suitable for initial clinical correction of arthrogrypotic clubfoot. Over the clinical course of this condition, surgeries will more likely become necessary in the more severely affected patients with AP. These findings can help physicians, caregivers and parents to plan and organise the treatment of these young patients. However, although the feet are among the most affected locations of arthrogryposis, with a proportion of about 80% [36], treatment of other aspects of the pathology in early childhood, as well as education on coping with the disease, must also be considered. The possibility of postponing more extensive surgical procedures can significantly boost a child’s overall development. The primary treatment goal in early childhood should be to enable children to learn to walk with orthoses. According to the results of this study and those in the literature, this can be achieved with the Ponseti method. It should be noted that the Ponseti method can only be linked to results if these were elevated close to its completion. Secondary surgical interventions are more likely to be a consequence of the type of initial surgical intervention and the natural course of the disease.

This study was limited by its retrospective nature and the small number of its included patients. According to our comprehensive literature review, the results of 144 patients concerning this topic have already been described in previous studies, and the number of our patients is about one-tenth of that. Moreover, this study did not account for other aspects that affect treatment success, including failure to follow bracing protocols. Real family compliance was not measured. At the last FU, only pain as a clinical parameter and the number of operations performed were recorded. Unfortunately, the Pirani score was not consistently documented in the medical records after completion of the first cast series and at the last FU. Recently published common data elements for AMC could not be considered [37]. Finally, radiological measurements were done only in lateral radiographs, especially for evaluating rocker bottom foot.

## Conclusion

The clinically high initial correction rate after Ponseti therapy for clubfoot with arthrogryposis was not accompanied by correction of the bony foot position in the X-rays. This was also reflected in the high rate of necessary corrective operations during the clinical course of the condition. The patients with DA had better outcomes than those with AP in all of the examined aspects. Therefore, in future outcome studies, a clear distinction between AP and DA patients is necessary. Initially high correction rates enable children with arthrogryposis to learn to walk without extensive surgery in early childhood, giving space for other important steps in child development. Furthermore, about a quarter of the patients benefited from Ponseti therapy without further major surgery in our nearly nine-year FU period.

## Electronic supplementary material

Below is the link to the electronic supplementary material.

Supplementary Material 1

Supplementary Material 2

Supplementary Material 3

## Data Availability

No datasets were generated or analysed during the current study.

## Abbreviations

AG: Arthrogrypotic
AMC: Arthrogryposis multiplex congenita
AP: Amyoplasia
CF: Complex foot reconstruction
DA: Distal arthrogryposis
DAA: Dorsal ankle arthrotomy
F: Female
FU: Follow-up
HE: Hindfoot equinus
IP: Idiopathic
L: Left
latTaCa: lateral talocalcaneal
latTaMT1: lateral talus-first metatarsal
m: male
n.a.: not available
n.m.: not mentioned
PTR: Peritalar release
r: right
RBF: Rocker bottom foot
reAT: repeat Achilles tenotomy
ROM: Range of motion
SDO: Supramalleolar derotational osteotomy
STR: Soft tissue release
TA: Triple arthrodesis
TATT: Tibialis anterior tendon transfer
TE: Talectomy
TSSA: Tibia shaft–sole angle
UCF: Under-corrected clubfoot
UHF: Under-corrected hindfoot
