# Supplementary material for: Midterm clinical and radiological outcomes of arthrogryposis-associated clubfoot treated with the Ponseti method: a retrospective observational study and comprehensive literature review
Source: J Orthop Surg Res. 2024 Sep 28;19:595. doi: 10.1186/s13018-024-05101-3 (PMC11437879; doi:10.1186/s13018-024-05101-3)
Supplement: Supplementary file 1 — Supplementary Material 1 [file 13018_2024_5101_MOESM1_ESM.docx]

**Supplementary Table 1** **Patient characteristics, clinical results and further surgeries**

| **Side** | **AP/DA** | **Gender** | **Initial Pirani score** | **Age at treatment start with cast** (weeks) | **Number of casts** | **Age at end of cast treatment** (weeks) | **Dorsiflexion after cast removal** (°) | **Clinical outcome after last cast removal** | **Radiological findings** | **Minor surgeries** | **Major surgeries** | **Age at minor surgeries** (years) | **Age at major surgeries** (years) | **Age at last FU** (years) | **Dorsi/plantarflexion at last FU** (°) |
| --- | --- | --- | --- | --- | --- | --- | --- | --- | --- | --- | --- | --- | --- | --- | --- |
| r | AP | m | 6 | 5.3 | 14 | 22.6 | -5 | poor | UCF | – | PTR | – | 0.8 | 6.0 | 5-0-10 |
| l |  |  | 6 |  | 14 |  | -5 | poor | UCF | – | PTR/CFR, SDO | – | 0.7/5.6 | 6.0 | 5-0-15 |
| r | DA1 | m | 6 | 3.1 | 14 | 19.4 | 10 | fair | UHF | – | PTR/TE | – | 1.9/9.2 | 10.4 | n.a. |
| l |  |  | 6 |  | 14 |  | 0 | fair | UHF | – | PTR | – | 2.1 | 10.4 | n.a. |
| r | DA1 | f | 5 | 2.0 | 10 | 15.1 | 10 | good | normal | – | – | – | – | 11.7 | 0-30-50 |
| l | DA3 | m | 6 | 2.7 | 10 | 17.1 | 15 | excellent | UHF | reAT, DAA, TATT | – | 1.8 | – | 7.7 | 10-0-5 |
| r | DA1 | m | 5 | 22.4 | 7 | 27.7 | 10 | excellent | UCF | – | – | – | – | 7.6 | 5-0-15 |
| l |  |  | 5 |  | 7 |  | 10 | excellent | UHF | – | – | – | – |  | 10-0-15 |
| l | AP | m | 4 | 7.9 | 12 | 18.9 | 10 | good | HE | – | TE | – | 6.1 | 10.8 | n.a. |
| r | DA1 | f | 6 | 7.0 | 21 | 27.4 | 0 | fair | HE | – | PTR/TA | – | 2.1/6.5 | 11.1 | 0-0-5 |
| l |  |  | 6 |  | 21 |  | 0 | fair | HE | – | PTR | – | 1.9 |  | 0-0-5 |
| r | AP | m | 4 | 4.3 | 6 | 11.1 | 15 | good | UCF | – | PTR/TA, SDO | – | 2.0/11.3 | 12.1 | 0-0-0 |
| l |  |  | 4 |  | 6 |  | 15 | good | UCF | reAT | TA | 2.0 | 6.7 |  | 0-0-0 |
| r | DA1 | f | 4.5 | 2.0 | 10 | 14.0 | 10 | good | HE | reAT | – | 1.1 | – | 5.2 | n.a. |
| l |  |  | 5 |  | 10 |  | 10 | good | HE | reAT | – | 1.1 | – |  | n.a. |
| r | AP | f | 5.5 | 21.4 | 12 | 28.7 | 10 | good | UHF | – | PTR | – | 2.1 | 9.4 | 5-5-0 |
| l |  |  | 5.5 |  | 12 |  | 10 | good | UHF | – | PTR | – | 2.1 |  | 5-5-0 |
| r | AP | f | 6 | 8.3 | 9 | 27.6 | -5 | poor | UHF | – | PTR/TE | – | 1.3/5.9 | 13.1 | 0-0-0 |
| l |  |  | 6 |  | 9 |  | -5 | poor | UHF | – | PTR/TE | – | 1.4/5.9 |  | 0-0-0 |
| r | DA1 | f | 6 | 1.3 | 11 | 11.0 | 20 | excellent | n.a. | – | – | – | – | 8.7 | 10-0-20 |
| l |  |  | 6 |  | 11 |  | 20 | excellent | n.a. | – | – | – | – |  | 10-0-20 |
| r | AP | m | 6 | 11.4 | 10 | 27.0 | 10 | good | RBF | – | TE | – | 4.3 | 8.5 | 5-0-5 |
| l |  |  | 6 |  | 10 |  | 10 | good | RBF | – | TE | – | 4.3 |  | 0-5-5 |
| r | AP | m | 6 | 22.6 | 6 | 28.3 | 0 | good | n.a. | – | PTR | – | 1.5 | 8.4 | 0-20-40 |
| l |  |  | 6 |  | 6 |  | 0 | good | n.a. | – | PTR | – | 1.4 |  | 0-30-30 |
| r | AP | m | 6 | 26.3 | 8 | 41.4 | 0 | good | UHF | reAT | PTR | 1.1 | 2.0 | 5.0 | 0-10-15 |
| l |  |  | 5 |  | 8 |  | 0 | good | UHF | reAT | PTR | 1.1 | 2.2 |  | 0-0-0 |
| r | DA4 | m | 6 | 3.0 | 12 | 16.7 | 0 | good | UCF | reAT | PTR | 1.0 | 1.7 | 7.4 | 0-5-15 |
| l |  |  | 6 |  | 12 |  | 0 | good | UCF | reAT | PTR | 1.0 | 1.7 |  | 0-5-10 |
| r | DA1 | m | 6 | 1.3 | 15 | 16.6 | 0 | good | UHF | – | TA | – | 7.0 | 11.1 | 5-0-5 |
| l |  |  | 6 |  | 15 |  | 5 | good | UHF | – | TA | – | 8.7 |  | 5-0-5 |
| r, right foot; l, left foot; AP, amyoplasia; DA, distal arthrogryposis; m, male; f, female; n.a., not available  HE, hindfoot equinus; UHF, under-corrected hindfoot; UCF, under-corrected clubfoot; RBF, rocker bottom foot;  Minor surgeries: reAT, Repeat Achilles tenotomy; DAA, dorsal ankle arthrotomy; TATT, tibialis anterior tendon transfer;  Major surgeries: PTR, peritalar release;CFR, complex foot reconstruction; SDO, supramalleolar derotational osteotomy; TE, talectomy; TA, triple arthrodesis | | | | | | | | | | | | | | | |
