# Supplementary material for: Midterm clinical and radiological outcomes of arthrogryposis-associated clubfoot treated with the Ponseti method: a retrospective observational study and comprehensive literature review
Source: J Orthop Surg Res. 2024 Sep 28;19:595. doi: 10.1186/s13018-024-05101-3 (PMC11437879; doi:10.1186/s13018-024-05101-3)
Supplement: Supplementary file 2 — Supplementary Material 2 [file 13018_2024_5101_MOESM2_ESM.docx]

**Supplementary Table 2 Radiological data of the treated feet**

| **Side** | **Age at X-ray** (years) | **Period cast removal – X-ray** (years) | **Clinical result** | **Radiological findings** | **Tibia shaft – sole angle** (°) | **LatTaMT1 angle** (°) **Negative ≙ pes cavus** [20] | **LatTaCa angle** (°) | **Calcaneal pitch** (°) |
| --- | --- | --- | --- | --- | --- | --- | --- | --- |
| r | 0.6 | 0.5 | poor | UCF | 132 | **-32** | **7** | **0** |
| l | 0.6 | 0.5 | poor | UCF | 108 | **-8** | **21** | **-6** |
| r | 0.6 | 0.3 | fair | UHF | 93 | 23 | **8** | **-24** |
| l | 0.6 | 0.3 | fair | UHF | 97 | 21 | **17** | **-16** |
| r | 6.8 | 6.5 | good | normal | 104 | -2* | 30* | 9* |
| l | 1.0 | 0.7 | excellent | UHF | 63 | 8 | **8** | **-20** |
| r | 1.2 | 0.7 | excellent | UCF | n.a. | **-17** | **0** | **5** |
| l | 1.2 | 0.7 | excellent | UHF | n.a. | 4 | **19** | **-3** |
| l | 1.8 | 1.4 | good | HE | 109 | 19 | 30 | **-11** |
| r | 0.8 | 0.3 | fair | HE | 107 | 24 | 36 | **-7** |
| l | 0.8 | 0.3 | fair | HE | 101 | 11 | 38 | **-5** |
| r | 1.8 | 1.5 | good | UCF | 114 | **-7** | **25** | **-5** |
| l | 1.8 | 1.5 | good | UCF | 94 | **-8** | **16** | **-12** |
| r | 1.1 | 0.8 | good | HE | n.a. | 8 | 28 | **-5** |
| l | 0.7 | 0.5 | good | HE | n.a. | 31 | 26 | **-12** |
| r | 1.8 | 1.2 | good | UHF | 99 | -1 | **10** | **-7** |
| l | 1.8 | 1.2 | good | UHF | 94 | 0 | **19** | **-13** |
| r | 1.1 | 0.6 | poor | UHF | 96 | 27 | **13** | **-17** |
| l | 1.1 | 0.6 | poor | UHF | 100 | 7 | **21** | **-13** |
| r | 3.5 | 3.0 | good | RBF | 86 | **37** | **17** | **-37** |
| l | 3.5 | 3.0 | good | RBF | 88 | **39** | **18** | **-31** |
| r | 0.7 | 0 | good | UHF | 89 | 0 | **4** | **-18** |
| l | 0.7 | 0 | good | UHF | 86 | 21 | **16** | **-27** |
| r | 1.4 | 1.1 | good | UCF | 120 | **-9** | **26** | **0** |
| l | 1.4 | 1.1 | good | UCF | 102 | **-5** | **22** | **-18** |
| r | 3.4 | 3.1 | good | UHF | 83 | 2 | **18** | **-8** |
| l | 2.9 | 3.1 | good | UHF | 86 | 0 | **28** | **-8** |
| - Pathological values in bold; *, borderline normal–pathological (latTaMT1) angle and (latTaCa) angle in relation to the age-dependent normal range accordingly (Vanderwilde et al.) [20] and calcaneal pitch accordingly (Davids et al.) with norm 17±6°) [21]. - The tibia shaft–sole angle reflects plantarflexion/dorsiflexion during X-ray. - Under-corrected clubfoot (UCF): pathological to small calcaneal pitch, pathological negative latTaMT1 angle, pathological to small latTaCa angle. - Under-corrected hindfoot (UHF): normal latTaMT1 angle, pathological calcaneal pitch and to small latTaCa angle. - Hindfoot equinus (HE): pathological to small calcaneal pitch. - Rocker bottom foot (RBF): pathological to small calcaneal pitch, pathological positive latTaMT1 angle, pathological to small latTaCa angle. | | | | | | | | |
