# Supplementary material for: Midterm clinical and radiological outcomes of arthrogryposis-associated clubfoot treated with the Ponseti method: a retrospective observational study and comprehensive literature review
Source: J Orthop Surg Res. 2024 Sep 28;19:595. doi: 10.1186/s13018-024-05101-3 (PMC11437879; doi:10.1186/s13018-024-05101-3)
Supplement: Supplementary file 3 — Supplementary Material 3 [file 13018_2024_5101_MOESM3_ESM.docx]

**Supplementary Table 3 Selected case series with outcomes of the Ponseti method in arthrogrypotic clubfeet**

| **Author/s, year, reference number** | **Location of institution** | **Number of patients** | **Data collection period** | **Median age at the treatment start** (years) | **Follow-up** (years) | **AP/DA/ unspecified/**  **other** | **Recurrence rate (AG/IP)** | **Endpoints** |
| --- | --- | --- | --- | --- | --- | --- | --- | --- |
| Schaibley et al., 2024 [25] | St. Louis, MO, USA | 18/334 (AG/IP) | 2002–2022 | 0.3/0.2 (AG/IP, *p* = 0.131) | 4.2±2.0/3.4±1.5 (AG/IP) | 6/12/0/0 | 83.3%/44.6% (*p* = 0.001); 91.7% DA vs 66.7% AP (*p* = 0.245),  surgery rate: 83.3/43.4% AG/IP | Recurrence, DA vs. AP subanalysis |
| **Conclusion:** At a three-year follow-up, the prognosis is less favourable. There are no significant differences in initial correction and recurrence rates between DA and AP. | | | | | | | | |
| Henstenburg et al., 2024 [26] | Philadelphia, USA; Brooklyn, USA; St. Louis, USA | 17 AG/30 feet | 2011–2019 | 2.31±1.54 | Study length, 5.9±1.1 | 6/4/6/1 | Only patients with a minimum of three casting series were included. | Improvement in Pirani scores |
| **Conclusion:** Clubfoot in AMC has a very high likelihood of recurrence. Early intervention with the Ponseti technique yields better results, with a diminished yet effective ability to elicit change over time. | | | | | | | | |
| Alam et al., 2023 [24] | Aligarh, India | 12 AG, 19 feet | 2019–2022 | 0.8 (0–3) | 1.83 (0.75–2.5) | n.m. | 42.1% | Initial correction (Pirani and Dimeglio), management of relapses |
| **Conclusion:** The initial outcome of the Ponseti technique in AMC is satisfactory. Relapses are higher than in classical idiopathic clubfoot cases, most of which respond to remanipulation and serial casting with retenotomy. | | | | | | | | |
| Church et al., 2020 [23] | Wilmington, DE, USA | 28/89 patients; 56/134 feet (AG/IP) | 2012–2019 | n.m. | 4.8±0.8 | n.m. | Further surgeries in 50% of the AG patients, 36% in IP, residual equinovarus in AG and IP | Passive range of motion, foot pressure analysis, Gross Motor Function Measure Dimension-D, parent report using the Pediatric Outcomes Data Collection Instrument |
| **Conclusion:** Despite some level of residual deformity, in children with clubfoot associated with arthrogryposis, the Ponseti method is successful in achieving a braceable foot that can delay the need for invasive surgical intervention. | | | | | | | | |
| Matar et al., 2016 [27] | Liverpool, UK | 10 AG/17 feet | 2005–2012 | 0.1 (0.04–0.4) | 5.8 (3.0–8.0) | n.m. | Recurrence in 50% of the patients, satisfactory outcome in 64.7% | Functional correction of the deformity (functional plantigrade, pain-free feet) |
| **Conclusion:** The Ponseti technique is an effective first-line treatment for arthrogrypotic clubfoot to achieve functional plantigrade feet. However, more casts are required, and there is a higher risk of relapse. | | | | | | | | |
| Ayadi et al., 2015 [22] | Sfax, Tunisia | 7 AG/12 feet | 2000–2012 | n.m. | n.m. | n.m. | 66.7% | n.m. |
| **Remark:** The study gave an overview of the clinical manifestations and management of arthrogryposis in 23 patients instead of focusing only on the use of the Ponseti method for clubfeet. | | | | | | | | |
| Kowalczyk et al., 2015 [28] | Krakow, Poland | 9 AG/18 feet | n.m. | 0.3 (0.2–0.4) | 7.3 (5–10) | AP | Good results in 14 patients, satisfactory results in 4; complication rate, 5.5%; reoperation rate, 1.2 per foot | Clinical outcome, anaesthesia and surgery times, reoperation rates |
| **Main findings/remark:** The Ponseti-treated patients were compared with 20 patients/39 feet initially treated with wide STRs. The primary STR showed a longer period of satisfactory correction. There were no differences in the total reoperation rates, but there was a higher rate of ablative and salvage reoperation in the STR group. Shorter anaesthesia and surgery times were reported in the Ponseti group. | | | | | | | | |
| Van Bosse et al., 2009 [29] | Philadelphia, PA, USA | 10 AG/19 feet | 2001–2006 | 1.4 (0.3–3.3) | 3.2 (1.1–5.8) | No DA | All feet remained braceable; ambulatory ability was not compromised; avoidance of extensive surgery until the last FU; 4 feet early, 2 patients with late recurrences | Dimeglio and Pirani scores, maximum ankle dorsiflexion |
| **Conclusion/Remark:** The Ponseti technique made possible corrections without extensive surgery during infancy or early childhood. A modified Ponseti technique was applied (additional initial Achilles tenotomy before casting). The study provided an additional literature review of STRs and primary talectomies before the Ponseti era. | | | | | | | | |
| Boehm et al., 2008 [10] | St. Louis, MO, USA; Hamburg, Germany | 12 AG/24 feet | n.m. | 0.3±0.4 | Minimum of 2 years | 12 DA (7 DA1, 3 DA2A, 1 DA4, 1 DA5) | Relapse in 6 feet of 3 patients (25%); Extensive STR in 1 patient | Dimeglio, number of necessary casts, relapse, necessary surgeries |
| **Conclusion:** The early-term results supported the use of the Ponseti method for the initial treatment of distal arthrogrypotic clubfoot deformity. | | | | | | | | |
| Kowalczyk et al., 2008 [11] | Kraków, Poland | 5 AG/10 feet | 2003–2007 | 3.0 (2.2–4.0) at FU | 3.0 (2.0–3.7) | 4/1/0/0 | 3 feet unsatisfactory (RBF in 2 feet, recurrence in 1 foot); additional surgical procedures in 9 feet | A plantigrade, pain-free and braceable foot was considered a satisfactory result; radiographic measurements: talocalcaneal angle, talometatarsal-first angle, tibiocalcaneal angle |
| **Conclusion:** There was an initial response to the Ponseti method, so it is an alternative for the initial treatment. However, there is a need for additional surgical intervention during the course of the condition. | | | | | | | | |
| Morcuende et al., 2008 [12] | St. Louis, MO, USA | 16 AG/32 feet | 1992–2004 | 0.3 (0–1) | 4.6 (0.8–12.1) | Upper and lower extremity involvement in 9 patients | Initial correction in 15 patients, relapse in 25%, no talectomies | Initial correction, relapse, need for surgical releases |
| **Conclusion:** The Ponseti method is very effective for initial correction. Subsequent surgeries are less extensive. | | | | | | | | |
| AP, amyoplasia; DA, distal arthrogryposis; AG, arthrogrypotic; IP, idiopathic; n.m., not mentioned; STR, soft tissue release; FU, follow-up | | | | | | | | |
